# Effects of acoustic complexity on growth performance, behavioral responses, and waterborne cortisol dynamics in Nile tilapia (*Oreochromis niloticus*)

**DOI:** 10.14202/vetworld.2026.1999-2009

**Published:** 2025-05-16

**Authors:** Hadiana Hadiana, Lee Seong Wei, Sholeh Hadi Pramono, Anik Martinah Hariati, Fadli Mulyadi, Abdillah Febri Awlarijal, Achmad Aprianto

**Affiliations:** 1 PSDKU Aquaculture, Faculty of Fisheries and Marine Sciences, Brawijaya University, Kediri City, East Java, Indonesia; 2 Department of Agro Industry, Faculty of Agro Industry and Natural Resources, University Malaysia Kelantan, Kota Bharu, Kelantan, Malaysia; 3 Electrical Engineering, Faculty of Engineering, Brawijaya University, Malang City, East Java, Indonesia; 4 Department of Fisheries and Marine Resources Management, Faculty of Fisheries and Marine Sciences, Brawijaya University, Malang City, East Java, Indonesia; 5 Department of Socio-Economic Agriculture, Faculty of Agriculture, Brawijaya University, Kediri City, East Java, Indonesia

**Keywords:** acoustic complexity, auditory enrichment, behavioral plasticity, growth performance, Nile tilapia, stress physiology, sustainable aquaculture, waterborne cortisol

## Abstract

**Background and Aim:**

Aquaculture intensification demands strategies that enhance productivity without compromising animal welfare. Auditory enrichment has emerged as a novel environmental intervention; however, the role of acoustic complexity in modulating physiological stress, behavior, and growth remains insufficiently explored. This study evaluated the effects of distinct musical genres on welfare and production performance in *Oreochromis niloticus*.

**Materials and Methods:**

A total of 75 juvenile *O. niloticus* were randomly assigned to five groups: Classical, Rock, Pop, Electronic (EDM), and Control (no music). Fish were exposed to 70 dB acoustic stimuli for 4 h daily over 30 days. Behavioral responses were assessed using ethograms and the Novel Tank Test, while stress was quantified non-invasively through waterborne cortisol analysis. Growth performance was evaluated using final weight, relative growth rate, specific growth rate, and survival. Statistical analysis was conducted using one-way analysis of variance with significance set at p < 0.05.

**Results:**

Acoustic treatments significantly influenced stress physiology, behavior, and growth. High-tempo genres (Rock and Electronic) induced chronic stress, reflected by elevated cortisol levels (up to 0.90 ng g^−1^ h^−1^), increased aggression and erratic swimming, reduced specific growth rate (1.2% day^−1^), and lower survival (67%). In contrast, Classical and Pop music promoted welfare, with reduced anxiety-like behaviors and stabilized cortisol profiles. The Pop group exhibited the highest growth performance, achieving superior final weight (13.67 ± 1.15 g), relative growth rate (142 ± 23%), and 100% survival. Behavioral observations suggested a unique “attentive immobility” state in the Pop group, potentially minimizing energy expenditure and enhancing metabolic allocation toward growth.

**Conclusion:**

Acoustic complexity is a critical and manageable environmental factor influencing welfare and productivity in *O. niloticus*. While high-intensity sound acts as a stressor, structured auditory stimuli, particularly Pop and Classical music, function as effective enrichment tools. These findings support the integration of optimized auditory protocols as a low-cost, non-invasive strategy for sustainable aquaculture intensification.

## INTRODUCTION

Global food security increasingly relies on aquaculture, yet the sector’s intensification faces a critical bottleneck: the trade-off between production efficiency and animal welfare [1]. In high-density systems, stressors such as poor water quality, overcrowding, and handling significantly impair immune function and growth performance, leading to economic losses [2, 3]. This challenge is particularly evident in Nile tilapia (*Oreochromis niloticus*), a cornerstone species of tropical aquaculture valued for its adaptability [4]. Despite its robustness, *O. niloticus* in intensive monoculture systems remains highly susceptible to chronic stress, which disrupts the hypothalamic–pituitary–interregnal axis and elevates cortisol levels, thereby suppressing somatic growth and feed conversion efficiency [5, 6]. Consequently, the development of non-invasive and cost-effective environmental enrichment strategies is essential for sustainable aquaculture intensification [8].

Environmental enrichment, traditionally focused on physical or social modifications, has recently expanded to include sensory domains, particularly auditory stimulation [7]. The aquatic environment is inherently rich in acoustic signals; however, the deliberate application of structured auditory enrichment remains a relatively unexplored frontier in aquaculture systems [8]. Studies in vertebrate models have demonstrated that rhythmic auditory stimulation can modulate physiological and behavioral states, yet aquatic applications have primarily focused on ornamental species [9, 10]. Although specific sound frequencies have been reported to induce calming or stimulatory effects [11], the influence of complex acoustic patterns, such as those represented by distinct musical genres, on the physiology and behavior of cichlid species remains poorly understood.

Despite increasing interest in auditory enrichment, several critical gaps remain in the current body of knowledge. First, most existing studies have focused on simple acoustic stimuli, such as single-frequency tones or generalized sound exposure, rather than evaluating the multidimensional complexity of sound, including tempo, rhythm, and frequency variation inherent in musical genres. Second, research on aquaculture species, particularly *O. niloticus*, remains limited, with most findings derived from ornamental or laboratory model fish, thereby limiting direct applicability to commercial production systems. Third, the integrative effects of acoustic complexity on multiple biological endpoints, namely stress physiology, behavioral responses, and growth performance, have not been systematically investigated within a unified experimental framework. Finally, the mechanistic relationship between acoustic stimuli and metabolic energy allocation, which may influence growth efficiency under stress conditions, remains largely unexplored. These limitations highlight the need for comprehensive studies that evaluate genre-specific acoustic effects in production-relevant aquaculture species.

Therefore, this study aims to evaluate the effects of auditory enrichment, characterized by distinct musical genres, on the welfare and production performance of *O. niloticus*. Specifically, the study investigates the influence of acoustic complexity on stress physiology through cortisol regulation, behavioral plasticity using the Novel Tank Test (NTT), and growth performance indicators, including weight gain and feed utilization efficiency. By integrating physiological, behavioral, and production metrics, this study seeks to establish a scientific basis for “acoustic welfare” as an innovative and scalable environmental management strategy. The findings are expected to provide practical insights for optimizing aquaculture systems through non-invasive auditory interventions, thereby enhancing both productivity and animal welfare in intensive farming conditions.

## MATERIALS AND METHODS

### Ethical approval

All experimental procedures involving juvenile *O. niloticus* were reviewed and approved by the Ethical Commission of Universitas Brawijaya, Indonesia (Protocol No. 023-KEP-UB-2024). The study was conducted in accordance with institutional animal care and use requirements and the Guidelines for the Treatment of Animals in Behavioral Research and Teaching issued by the Animal Behavior Society.

Before the experiment, fish were acclimatized under controlled laboratory conditions to minimize handling stress. During the study, fish were maintained under optimal water quality, stocking density, temperature, and photoperiod conditions appropriate for *O. niloticus*. All procedures were designed to minimize pain, distress, and unnecessary disturbance. Behavioral observations and waterborne cortisol sampling were performed using non-invasive or minimally invasive approaches wherever possible.

Humane endpoints were established before the start of the experiment. Fish showing persistent loss of equilibrium, severe erratic swimming, prolonged unresponsiveness, marked respiratory distress, or inability to maintain normal swimming posture were to be removed immediately from the experiment. When euthanasia was required, fish were humanely euthanized using an overdose of tricaine methanesulfonate (MS-222; Sigma-Aldrich, St. Louis, MO, USA), followed by confirmation of death before disposal. A total of 75 fish were approved for use under this protocol.

### Study period and location

The experiment was conducted at the aquaculture research facility of Universitas Brawijaya, East Java, Indonesia. The study was performed under controlled laboratory conditions with standardized environmental parameters throughout the experimental period.

### Study design

Juvenile *O. niloticus* (N = 75; initial mean weight: 4.80 ± 0.50 g, range: 4.2–5.5 g) were obtained from a commercial hatchery certified free of common pathogens and acclimatized for 1 week before the experiment. A one-way analysis of variance (ANOVA) confirmed no significant differences in initial body weight among groups at baseline (p > 0.05). Fish were randomly distributed among 15 glass aquaria (10 L capacity), resulting in an initial stocking density of approximately 2.4 g/L, which reached a maximum of 6.8 g/L at the end of the trial.

Rearing conditions were maintained at optimal levels for *O. niloticus*. Water temperature was thermostatically controlled at 28°C ± 1°C. A defined photoperiod of 12 h light:12 h dark was maintained. Water quality parameters, including dissolved oxygen, pH, and ammonia-nitrogen, were monitored daily. To minimize background noise and vibrational interference, filtration was managed using a biological sponge filter supplemented with a microbial consortium (nitrifying and photosynthetic bacteria) to facilitate biofiltration, with partial water exchanges (20%) performed every 48 h.

### Acoustic enrichment setup

The experimental design consisted of five treatments defined by the acoustic environment:

- T1 (Classical): Beethoven/Mozart (60–70 beats per min)
- T2 (Rock): Metallica/Nirvana (100–140 beats per min)
- T3 (Pop): Ed Sheeran/Taylor Swift (90–120 beats per min)
- T4 (Electronic): Electronic dance music/Lo-Fi (synthesized, repetitive rhythm)
- T5 (Control): ambient facility noise only

Sound transmission was delivered via submerged underwater transducers (Model Stw-c, 8 Ω, 1.5 W; Shenzhen Electronics Co., Ltd., Shenzhen, China), positioned 5 cm below the water surface and isolated from tank walls to prevent vibro-acoustic distortion. Sound pressure levels were calibrated to 70 dB (re 1 μPa) using a HTI-96-MIN hydrophone (High Tech Inc., Gulfport, MS, USA), measured at the center and corners of each aquarium (variability ± 2 dB).

To avoid pseudoreplication, treatments utilized randomized looping playlists of 2–3 representative tracks (e.g., Beethoven’s Symphony No. 5 for Classical). Future objective acoustic characterization (dominant frequency bands, spectral centroid, and amplitude modulation) will further define the acoustic complexity driving these genre-specific responses.

**Figure 1:**
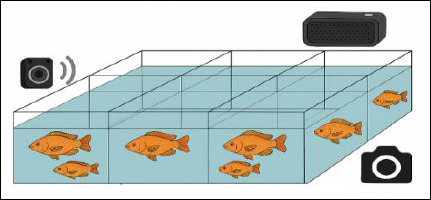
Schematic representation of the fish management research system.

Auditory enrichment was provided for a total of 4 h daily, divided into two sessions (09:00–11:00 and 15:00–17:00). The stimulus consisted of a 30-min looping playlist. To prevent habituation, a washout period (silence) was implemented for 1 week between distinct experimental phases. Control groups were acoustically isolated using sound-dampening partitions. The experimental setup included a schematic representation of the fish management research system, as shown in Figure 1.

### Behavioral analysis

**Figure 2:**
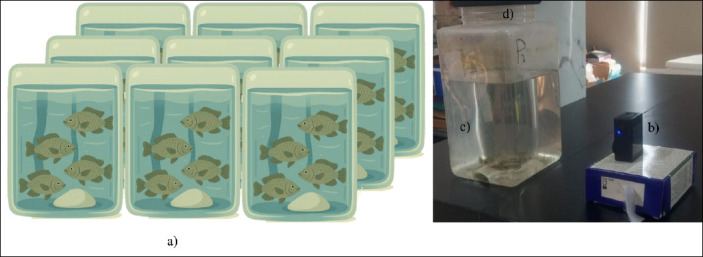
Schematic diagram of one music enrichment group and a partial view of the experimental setup: (a) experimental group, (b) camera, (c) aquarium tank of *Oreochromis niloticus*, and (d) speaker.

Behavioral plasticity was assessed on days 1, 3, and 5 of the enrichment periods. High-definition cameras (B-Pro 5 Alpha Edition, Brica Corp., Jakarta, Indonesia) recorded fish activity at 5-min intervals during the stimulation periods (Figure 2). Videos were analyzed blindly by two independent observers using the ethogram adapted from [12].

### NTT

**Figure 3:**
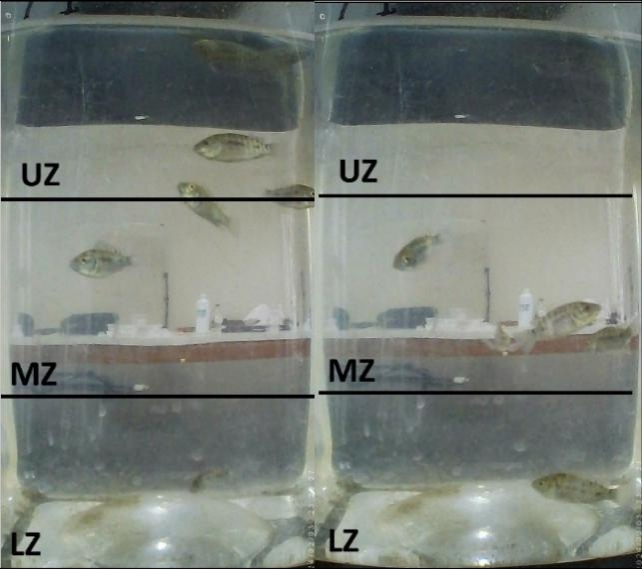
Schematic representation of the experimental arrangement for NTT, illustrating the predefined zones: upper zone (UZ), middle zone (MZ), and lower zone (LZ).

To assess anxiety-related behavior, NTT was conducted post-experiment (Day 30). A dedicated observation tank, geometrically identical to rearing tanks, was visually divided into three vertical zones: upper zone (UZ), middle zone (MZ), and lower zone (LZ). Fish (n = 6 per treatment) were individually introduced into the NTT apparatus (Figure 3).

Videos were analyzed by two independent observers blinded to treatment groups using the ethogram (Table 1), achieving an inter-observer reliability of Cohen’s kappa > 0.80. A 10-min acclimatization period was used before a 3-min recording. Time spent in the upper zone was interpreted as high exploratory activity (low-anxiety), whereas thigmotaxis (bottom-dwelling) in the lower zone indicated high-anxiety.

**Table 1:** Behavior spectrum of *Oreochromis niloticus*.

Behavioral category	Behavior	Description
Positive (good welfare state)	Touch water	Contact with the water surface or aquarium walls using the head or caudal fins, indicating exploratory behavior and favorable welfare
	Follow	Persistent tracking of a conspecific along a defined trajectory, indicating active social interaction
	Wander	Slow, repetitive swimming within a restricted spatial range, reflecting stable behavioral expression
Negative (poor welfare state)	Freezing	Prolonged immobility (>5 s), indicating anxiety or fear response
	Aggression	Repetitive localized swimming used to displace conspecifics, indicating dominance behavior
	Sprint	Abrupt acceleration or directional change without stimulus, indicating stress or agitation
Novel Tank Test	Upper dwell time	Occupation of the upper water column, indicating low-anxiety and exploratory behavior
	Lower dwell time	Residence near the bottom, indicating high-anxiety

### Waterborne cortisol quantification

To assess physiological stress responses without inducing handling stress, cortisol levels were measured via waterborne sampling using the non-invasive protocol previously described [12]. At the end of the experimental period, fish (n = 6 per treatment) were individually transferred into static glass beakers containing 250 mL of system water (28°C).

Fish were confined for 30 min to allow passive diffusion of free cortisol across the gill epithelium. After confinement, fish were returned to rearing tanks, and water samples (5 mL) were immediately collected and stored at −20°C. Cortisol concentrations were quantified using a species-validated enzyme-linked immunosorbent assay kit (MM-008901; Jiangsu Meimian Industrial Co., Jiangsu, China). The intra- and inter-assay coefficients of variation were <10%.

To prevent habituation bias from longitudinal sampling, independent subsets of fish (n = 6 per treatment) were sampled at each weekly time point [13]. Optical density was measured at 450 nm, and values were normalized to fish wet weight and confinement duration, expressed as ng g^−1^ h^−1^ [14].

### Statistical analysis

Data were tested for normality using the Shapiro–Wilk test and homogeneity of variance using Levene’s test. Differences among acoustic treatments were analyzed using one-way ANOVA. Significant main effects were followed by Fisher’s least significant difference post hoc tests. All analyses were performed using IBM SPSS Statistics (version 25.0; IBM Corp., Armonk, NY, USA), with significance established at p < 0.05. Data are presented as mean ± SEM.

## RESULTS

### Waterborne cortisol response

Physiological stress responses, quantified via waterborne cortisol, exhibited divergent trajectories across acoustic treatments (Table 2). Fish exposed to high-tempo, complex acoustic stimuli (Rock and Electronic) demonstrated a progressive elevation in cortisol concentrations, culminating in peak levels by Week 4 (0.90 ng g^−1^ h^−1^). Conversely, the Control and Classical groups exhibited a habituation pattern, characterized by an initial fluctuation in Week 2 followed by stabilization or decline in cortisol levels by Week 4 (0.075 ng g^−1^ h^−1^). The Pop music group maintained intermediate, stable cortisol concentrations (0.45 ng g^−1^ h^−1^) from Week 2 through Week 4.

**Table 2:** Cortisol levels in *Oreochromis niloticus* during 4 weeks.

Treatment	Week 1 (ng g⁻¹ h⁻¹)	Week 2 (ng g⁻¹ h⁻¹)	Week 3 (ng g⁻¹ h⁻¹)	Week 4 (ng g⁻¹ h⁻¹)
Control	0.045 ± 0.005ᵃ	0.450 ± 0.042ᵃ	0.150 ± 0.012ᵃ	0.075 ± 0.008ᵃ
Classical	0.075 ± 0.008ᵃ	0.300 ± 0.028ᵃ	0.150 ± 0.015ᵃ	0.075 ± 0.009ᵃ
Pop	0.075 ± 0.006ᵃ	0.450 ± 0.050ᵃᵇ	0.450 ± 0.041ᵇ	0.450 ± 0.038ᵇ
Rock	0.150 ± 0.012ᵇ	0.600 ± 0.055ᵇ	0.750 ± 0.068ᶜ	0.900 ± 0.082ᶜ
Electronic	0.150 ± 0.015ᵇ	0.600 ± 0.062ᵇ	0.750 ± 0.071ᶜ	0.900 ± 0.085ᶜ

### Behavioral plasticity and welfare indicators

**Figure 4:**
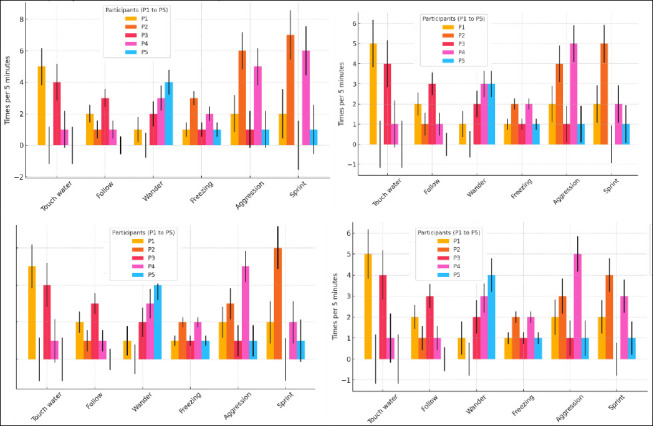
Effects of music enrichment on the behavior of *Oreochromis niloticus*. Different superscript letters within the same behavioral category denote statistically significant differences among groups (p < 0.05).

**Ethogram analysis:** Acoustic enrichment significantly modulated activity patterns (p < 0.05; Figure 4). Fast-tempo genres (Electronic dance music and Pop) were associated with elevated frequencies of stress-related behaviors. Specifically, the Electronic group (P4) exhibited significantly higher rates of sprinting and aggression compared to the Control (P5) and Classical (P1) groups, particularly during the acclimatization phase (Weeks 1–2; p < 0.05).

While freezing behavior was initially elevated in the Pop (P3) and Electronic (P4) groups, the Classical (P1) and Control (P5) groups displayed behavioral stability, characterized by significantly higher frequencies of wandering (a steady-state behavior) by Week 3 (p < 0.05).

**Figure 5:**
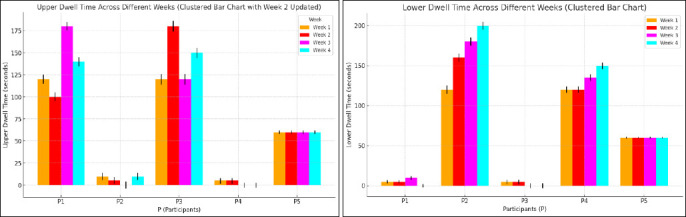
Effects of music enrichment on Novel Tank Test performance in *Oreochromis niloticus*: (a) upper dwell time and (b) lower dwell time.

**NTT:** NTT revealed distinct and music-specific behavioral phenotypes, providing a robust measure of anxiety-like states and exploratory drive (Figure 5). Exposure to Rock (P2) and Electronic (P4) music induced a high-anxiety thigmotactic profile, evidenced by a significantly greater proportion of time spent in the LZ compared to the Pop (P3) and Classical (P1) groups (p < 0.01). This persistent bottom-dwelling behavior is widely interpreted as an indicator of behavioral inhibition and elevated anxiety.

Conversely, fish exposed to Pop (P3) and Classical (P1) music exhibited a low-anxiety/exploratory phenotype, characterized by significantly increased dwell time in the UZ during the later phase of the test (Weeks 3 and 4; p < 0.05). This preference for the UZ indicates reduced anxiety and enhanced exploratory behavior, suggesting that these auditory stimuli promote adaptive responses to novel environments.

### Growth performance and feed utilization

**Figure 6:**
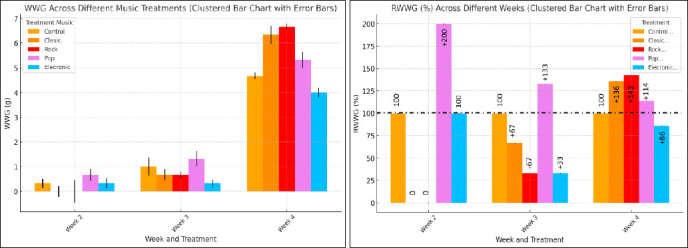
Wet weight gain and relative wet weight gain of *Oreochromis niloticus* exposed to different acoustic environments for 30 days. Data are presented as mean ± SEM. Bars with different letters indicate significant differences among groups (p < 0.05).

Somatic growth metrics were significantly influenced by the acoustic environment after the 30-day exposure period (*p* < 0.05; Table 3). Exposure to specific music paradigms significantly affected weight gain and Relative Growth Rate. The Pop group (P3) exhibited the most pronounced anabolic response, achieving the highest final weight (13.67 ± 1.15 g) and superior RGR (142 ± 23%), showing a significant advantage over the Rock and Electronic groups (p < 0.05) (Figure 6).

**Table 3:** Growth performance and feed utilization of *Oreochromis niloticus* following 30 days of acoustic enrichment.

Parameter	Control	Classical	Rock	Pop	Electronic
ITW (g)	5.00 ± 1.00	4.00 ± 1.00	4.67 ± 0.58	5.67 ± 0.58	4.67 ± 0.58
FW (g)	10.33 ± 2.31	12.67 ± 0.58	8.33 ± 0.58	13.67 ± 1.15	9.33 ± 0.58
RGR (%)	118 ± 94	136 ± 97	80 ± 20	142 ± 23	102 ± 23
FCR	0.19 ± 0.02	0.19 ± 0.02	0.19 ± 0.01	0.19 ± 0.02	0.20 ± 0.01
Survival (%)	93	87	67	100	80

This observation suggests that reduced anxiety and enhanced exploratory behavior in the Pop group, as observed in NTT, contributed to improved metabolic efficiency and nutrient assimilation. The Classical group (P1) also demonstrated enhanced growth performance, with RGR (136 ± 97%) exceeding that of the Control group, indicating beneficial physiological effects of structured auditory stimuli.

**Figure 7:**
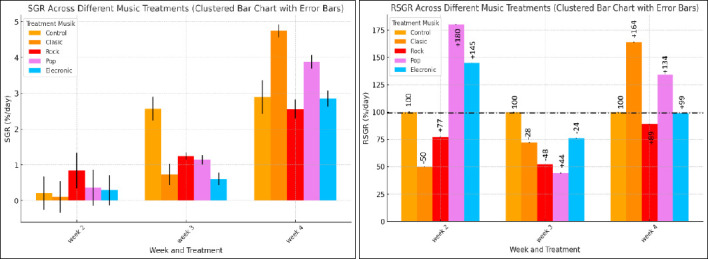
Specific growth rate and relative specific growth rate of *Oreochromis niloticus* under different acoustic conditions. Data are presented as mean ± SEM. Bars with different letters indicate significant differences among groups (p < 0.05).

Analysis of daily growth efficiency revealed a significant hierarchy among treatments (p < 0.05; Figure 7. The Pop group (P3) achieved the highest Specific growth rate (3.5% day^−1^), followed by the Classical group (3.0% day^−1^), both significantly higher than the Control group (2.5% day^−1^).

In contrast, exposure to Rock (P2) and Electronic (P4) stimuli resulted in significant growth suppression. The Rock group exhibited the lowest SGR (1.2% day^−1^), representing a 52% reduction compared to the Control group. These findings indicate that while Pop and Classical acoustic environments function as growth-promoting conditions, high-intensity acoustic stimuli act as metabolic stressors.

The differential effects of auditory stimuli were further reflected in survival and resource utilization efficiency. The Pop group (P3) achieved 100% survival, whereas the Rock group (P2) exhibited the lowest survival rate (67%). Mortalities in the Rock group occurred predominantly during the final 2 weeks. Necropsy observations revealed no signs of secondary pathogenic infection, indicating that mortality was likely due to chronic acoustic stress-induced exhaustion.

Feed conversion ratio remained relatively consistent across treatments (mean ≈ 0.19). However, the Electronic group (P4) showed a slight, non-significant increase (0.20 ± 0.01), suggesting reduced feed utilization efficiency associated with stress-related behavioral patterns.

## DISCUSSION

### Acoustic environment as a regulator of stress and growth

The present study provides empirical evidence that the acoustic environment is a potent modulator of physiological homeostasis and somatic growth in juvenile *O. niloticus*. This study is among the first to systematically compare four distinct musical genres, including high-tempo and complex profiles such as Rock and Electronic dance music, against traditional Classical benchmarks in a production-relevant cichlid species. Previous auditory enrichment studies in aquaculture have largely focused on cyprinids or salmonids using single-frequency tones or Classical music; however, the present findings demonstrate that the structural properties of complex acoustic stimuli critically govern stress-coping capacity and growth efficiency in *O. niloticus*. High-intensity, fast-tempo acoustic profiles (Rock and Electronic) functioned as chronic stressors, whereas predictable and rhythmic stimuli (Classical and Pop) acted as effective welfare-enhancing conditions.

### Acoustic stress and metabolic dysregulation

The physiological maladaptation observed in the Rock (P2) and Electronic (P4) groups supports the acoustic stress hypothesis. These groups exhibited the highest waterborne cortisol concentrations and the most pronounced stress-related behaviors, including sustained sprinting and aggression. Similar physiological responses have been documented in aquatic species exposed to high-intensity or irregular acoustic environments, which resulted in dysregulated stress responses and elevated cortisol levels in koi carp [15, 16]. Sustained activation of the hypothalamic–pituitary–interregnal axis under such aversive conditions likely maintained a catabolic physiological state, diverting energy away from anabolic processes such as growth toward essential survival mechanisms [17, 18]. This energy trade-off was reflected in the markedly reduced SGR and survival rate (67%) observed in the Rock group.

In contrast, reduced cortisol concentrations in the Classical and Pop groups align with previous observations in zebrafish and carp, where structured and melodic acoustic stimuli attenuated endocrine stress responses [19, 20]. The sensitivity of *O. niloticus* to acoustic cues has been well documented [21], emphasizing the importance of predictable and non-threatening auditory environments in maintaining physiological stability.

### Growth performance and metabolic allocation theory

Acoustic complexity exerted a significant influence on production outcomes. The detrimental impact of high-tempo acoustic stimuli on growth, particularly the low SGR (1.2% day^−1^) observed in the Rock group, is consistent with previous findings linking acoustic stress to impaired growth efficiency in aquaculture species [22, 23]. In contrast, both Classical and Pop treatments promoted growth, with the Pop group achieving the highest SGR (3.5% day^−1^).

A key contribution of this study lies in the identification of a distinct behavioral–metabolic pattern in the Pop group. Despite exhibiting frequent freezing behavior, this group achieved maximal growth performance and 100% survival. This observation, referred to here as a behavioral–metabolic dissociation, challenges the conventional interpretation of freezing behavior as a purely stress-induced response [26, 27]. Freezing is typically associated with fear and anxiety in fish behavioral paradigms [28]; however, the absence of elevated anxiety indicators in NTT and the lack of mortality suggest an alternative functional interpretation.

It is proposed that this immobility reflects a state of attentive immobility or passive auditory engagement rather than maladaptive stress [29]. Under this framework, the Pop group may have minimized locomotor energy expenditure compared to the energetically demanding sprinting behavior observed in the Rock group. This reduction in energy expenditure could facilitate a more efficient allocation of metabolic resources toward somatic growth, consistent with the principles of metabolic allocation theory [30, 31].

The observed SGR of 3.5% day^−1^ exceeds commonly reported values for *O. niloticus* juveniles (1–2.5% day^−1^), suggesting that optimized environmental conditions combined with structured auditory enrichment may synergistically enhance growth performance [25]. However, direct physiological validation, such as measurements of oxygen consumption or proximate body composition, is required to confirm this proposed mechanism [32].

### Implications for aquaculture systems and future directions

These findings establish the auditory environment as a critical yet manageable determinant of *O. niloticus* welfare and productivity. By challenging the conventional reliance on Classical music in aquaculture research, the present study demonstrates that contemporary acoustic profiles, particularly Pop, can produce equal or superior benefits.

The application of low-cost, genre-specific auditory enrichment protocols represents a practical bioengineering strategy for improving productivity in commercial systems, including Recirculating Aquaculture Systems and intensive pond-based operations. Such interventions offer a non-invasive approach to optimizing stress regulation and metabolic efficiency without requiring structural modifications to existing systems.

Future research should focus on quantifying the acoustic parameters responsible for these effects, including amplitude modulation, frequency distribution, and spectral complexity. Additionally, integrating physiological measurements such as metabolic rate, oxygen consumption, and nutrient partitioning will be essential to elucidate the mechanistic basis of the observed responses.

## CONCLUSION

The present study demonstrates that acoustic complexity is a decisive environmental factor regulating stress physiology, behavior, and growth performance in juvenile *O. niloticus*. Exposure to high-intensity, fast-tempo acoustic environments (Rock and Electronic) resulted in elevated cortisol concentrations (up to 0.90 ng g^−1^ h^−1^), increased stress-related behaviors (aggression and sprinting), reduced SGR (as low as 1.2% day^−1^), and decreased survival (67%). In contrast, structured and rhythmic auditory stimuli, particularly Pop and Classical music, promoted physiological stability, reduced anxiety-like behavior in NTT, and enhanced production performance. The Pop group achieved the highest final weight (13.67 ± 1.15 g), superior RGR (142 ± 23%), maximal SGR (3.5% day^−1^), and 100% survival, indicating a strong association between acoustic environment and metabolic efficiency.

A key strength of this study lies in its integrative approach, simultaneously evaluating physiological (waterborne cortisol), behavioral (ethogram and NTT), and production (growth and feed utilization) parameters within a single experimental framework. The use of non-invasive stress assessment and production-relevant conditions enhances the applicability of the findings to commercial aquaculture systems. Additionally, the comparative evaluation of multiple musical genres provides novel insights into the role of acoustic complexity rather than simple sound exposure.

However, several limitations should be acknowledged. The study did not directly measure metabolic rate, oxygen consumption, or energy partitioning, which limits mechanistic interpretation of the observed growth responses. Acoustic parameters such as frequency spectra, amplitude modulation, and sound field uniformity were not quantitatively characterized in detail. Furthermore, the relatively short experimental duration and controlled laboratory conditions may not fully capture long-term or field-scale responses in commercial production systems.

Despite these limitations, the findings provide compelling evidence that carefully structured auditory environments can serve as an effective, low-cost, and non-invasive strategy to enhance welfare and productivity in aquaculture. The identification of a potential “attentive immobility” state associated with improved growth in the Pop treatment offers a novel perspective on behavioral–metabolic interactions in fish.

In conclusion, the integration of optimized auditory enrichment protocols, particularly those based on structured and rhythmic acoustic profiles, represents a promising approach for sustainable intensification of tilapia farming. Future studies incorporating detailed acoustic profiling and physiological measurements are warranted to refine these strategies and establish standardized guidelines for practical implementation.

## DATA AVAILABILITY

The data generated during the study are included in the manuscript.

## AUTHORS’ CONTRIBUTIONS

HH, LSW, and SHP: Conceptualization and study design. HH, AFA, and AA: Experimental work, behavioral observations, and waterborne cortisol sampling. AMH and FM: Statistical analyses. HH: Writing – original draft. SHP, AMH, FM, AFA, and AA: Critical review and editing of the manuscript for intellectual content. All authors have read and approved the final version of the manuscript.
